# Multi-View Image Denoising Using Convolutional Neural Network

**DOI:** 10.3390/s19112597

**Published:** 2019-06-07

**Authors:** Shiwei Zhou, Yu-Hen Hu, Hongrui Jiang

**Affiliations:** Department of Electrical and Computer Engineering, University of Wisconsin-Madison, Madison, WI 53706, USA; hongrui@engr.wisc.edu

**Keywords:** multi-view denoising, convolution neural network, 3D focus image stacks, disparity estimation

## Abstract

In this paper, we propose a novel multi-view image denoising algorithm based on convolutional neural network (MVCNN). Multi-view images are arranged into 3D focus image stacks (3DFIS) according to different disparities. The MVCNN is trained to process each 3DFIS and generate a denoised image stack that contains the recovered image information for regions of particular disparities. The denoised image stacks are then fused together to produce a denoised target view image using the estimated disparity map. Different from conventional multi-view denoising approaches that group similar patches first and then perform denoising on those patches, our CNN-based algorithm saves the effort of exhaustive patch searching and greatly reduces the computational time. In the proposed MVCNN, residual learning and batch normalization strategies are also used to enhance the denoising performance and accelerate the training process. Compared with the state-of-the-art single image and multi-view denoising algorithms, experiments show that the proposed CNN-based algorithm is a highly effective and efficient method in Gaussian denoising of multi-view images.

## 1. Introduction

Image denoising is an essential tool for image quality enhancement. It is often a required preprocessing step to facilitate effective image understanding and other computer vision tasks, such as segmentation, classification, and object detection. Due to the limitations of optical and electronic devices, noise is inevitable in the process of image capture, which can be described using the image degradation model *y* = *x* + *n*, where *x* is the clean image, *y* is the noisy observation, and *n* is the additive noise, which is often modeled as additive white Gaussian noise (AWGN). Though real-world noises are far more complicated, they can be approximated locally as AWGN [1], which is a natural choice when the prior information of the noise in question is unknown. The purpose of image denoising is to estimate *x*, given *y* and some statistical properties of *n*.

In recent years, with the increasing desire for 3D information that a single image cannot provide, multi-view imaging systems have acquired attention from researchers and commercial companies [2]. With multiple cameras capturing the same scene simultaneously from different viewpoints, disparities between distinct views can be acquired to recover the 3D information of the scene. However, cameras in multi-view systems usually have limited aperture and sensor size, which result in noise corruption in the captured images. In multi-view image denoising, the single image noise model is applied to each of the views, and our goal is to achieve an estimate of the target view given a number of noisy observations.

Conventional image denoising methods [3,4,5,6,7,8,9,10,11,12,13,14,15,16,17,18,19,20,21,22] attempt to exploit various kinds of models that can approximately describe the prior image. These model-based approaches, though capable of achieving state-of-the-art denoising performance, are generally computationally expensive due to exhaustive patch matching and optimization algorithms. Meanwhile, these models usually employ many handcrafted parameters that need to be determined heuristically in prior, which is not flexible enough to handle different image structures.

On the other hand, discriminative learning methods [23,24,25,26,27,28,29,30,31] have been recently developed to learn the image prior in a data-driven manner that does not involve manual design. The most successful among them is the convolutional neural network (CNN) that has a deep architecture for effectively exploiting the image characteristics. Training data with degraded and ground truth image pairs are fed into the network so that network parameters can be learned automatically with fast inference. However, most existing algorithms, model-based or learning-based, are designed for single image denoising. As far as we know, there is no existing deep network denoising algorithm developed for multi-view images.

In this work, we present a convolutional neural network for multi-view image denoising (MVCNN). Instead of using a single image as the input, the network we propose receives multiple views that have been preprocessed and formed as a 3D matrix. The network can predict the residual images which are also in the form of a 3D matrix. Subsequently, the denoised images are obtained by subtracting the residual images from the observed noisy images. In general, the contribution of this paper can be summarized below:

- A convolutional neural network that takes multiple views (in the form of 3D matrix) as the input and delivers multiple residual images as the output.
- An efficient image fusion approach that integrates multiple denoised 3D focus image stacks into a target denoised image using the disparity map.
- A novel and effective technique that detects and tackles occlusions from the disparity map through morphological transformations.

To form the input 3D matrix, the proposed algorithm uses a special image structure called 3D focus image stacks (3DFIS) that has been introduced in our previous work [32]. However, instead of searching for similar patches in the 3DFIS and performing denoising on the grouped patches as was done in [32], we process the entire image stacks through the proposed network and then fuse these denoised image stacks using the disparity map to obtain the final denoised image. This new processing of the 3DFIS using convolutional neural network helps us avoid the time-consuming patch searching procedure, and hence significantly reduces the computational time, in addition to the performance improvement.

The remainder of the paper is organized as follows. Section 2 gives a brief review of various image denoising algorithms, including single image and multi-view methods. Section 3 and Section 4 present the proposed MVCNN model and the corresponding denoising algorithm. Section 5 demonstrates the experimental results compared with current state-of-the-art approaches. Finally, the conclusion of the paper is given in Section 6.

## 2. Related Work

### 2.1. Conventional Image Denoising

Conventional denoising algorithms model image denoising as an inverse problem that can be approximated as maximum a posteriori (MAP) estimation using Bayesian inference. The problem may be solved by applying various optimization strategies based on the image prior modeling. Over the past decades, numerous image prior models have been proposed. One of the most popular models is the non-local self-similarity (NSS) [3,4,5,6,7,8,9], following the observation that a local patch has many non-local similar patches across the image. Many of the state-of-the-art algorithms employ this model, including Block-matching 3D (BM3D) [6] and Weighted Nuclear Norm Minimization (WNNM) [8]. Meanwhile, researchers have also explored various other models, such as Markov random field (MRF) [10,11,12,13], total variation [14,15,16,17,18], and sparsity [19,20,21,22]. Some of these methods also achieve great success in terms of denoising quality. However, the complex optimization and exhaustive patch matching have limited their applications in real-world problems due to the excessive computation burden involved. The manually and heuristically determined parameters also lack flexibility when image structures are abundant in real-world scenarios.

### 2.2. Deep Neural Networks for Single Image Denoising

Unlike conventional methods that learn the noise model using a specific statistical model with the requirement of well-designed prior, deep neural network approaches learn the mapping between noisy and clean images in a data-driven manner that achieves optimal denoising beyond human design. Barbu et al. [23] proposed to train an MRF model with a fast inference algorithm through optimization of a loss function on the training set. In [24], Xie et al. advocated adapting a denoising auto-encoder that was designed for unsupervised feature learning to image denoising tasks. Later, Schmidt et al. [25] put forward a random field-based architecture called shrinkage fields to effectively learn the model parameters. Inspired by the field-of-expert (FoE) based model [11], Chen et al. [27] further developed a trainable non-linear diffusion reaction (TNRD) algorithm that optimizes a time-discrete partial differential equation with gradient descent/forward-backward steps. While early methods cannot compete with state-of-the-art algorithms like BM3D, some of the recently developed algorithms, such as TNRD, have achieved competitive or even better denoising performance.

In the meantime, plain discriminative learning methods that do not require prior explicit modeling of an image have also received increasing attention. Burger et al. [27] learned a mapping between noisy and clean images directly with a plain multi-layer perceptron (MLP) applied to image patches. Recently, Zhang et al. [28] proposed a CNN-based network (DnCNN) that successfully adopts residual learning and batch normalization to image denoising problems. DnCNN also achieves the current state-of-the-art performance among learning-based algorithms that outperforms conventional approaches. Following the success of DnCNN, the same group later developed a more flexible FFDNet algorithm [29]. The algorithm aims to deal with spatially variant noise by introducing a noise level map and applying orthogonal regularization to improve the robustness to noise level mismatch. Jin et al. [30] proposed to use direct inversion followed by a CNN to solve general normal-convolutional inverse problems, including denoising. In order to improve the robustness and practicability of deep denoising models to real-world noise, Guo et al. [31] implemented a convolutional blind denoising network comprised of a noise estimation subnetwork and a denoising subnetwork. The network is trained using a more realistic noise model by considering both the signal-dependent noise and the in-camera processing pipeline.

### 2.3. Multi-View Image Denoising

In the field of multi-view denoising, inter-view image dependencies are used to facilitate similar patch matching, such that denoising performance can be further improved. Zhang et al. [33] proposed a principal component/tensor analysis based denoising algorithm using a depth-guided patch similarity measure. Similarly, Luo et al. [34] incorporated a depth-dependent robust metric in their adaptive non-local means algorithm. In the perspective of 3D reconstruction, Xue et al. [35] introduced a graphical model of surface patches that is able to model the intra-view and inter-view redundancy more effectively, and noise can be attenuated using Wiener filtering on the sparse representation of these patches. More recently, Yue et al. [36] employed a two-stage strategy that explores both internal and external correlations with the help of web images. To accelerate processing speed, Miyata et al. [37] developed a fast multi-view image reconstruction algorithm. This algorithm uses plane sweeping [38] to obtain a number of pre-denoised images and assembles the in-focus parts of those images to get the final estimation. Inspired by plane sweeping, our previous work [32] introduced a new data structure called 3D focus image stacks (3DFIS) and a more robust multi-view denoising algorithm that incorporates depth-guided adaptive windows and low-rank approximation.

Recently, the application of a deep neural network to multi-view denoising has attracted researchers’ attention. Chen et al. [39] proposed a light field denoising framework based on anisotropic parallax analysis. In this work, two convolutional neural networks (CNN) will jointly predict parallax information and restore non-Lambertian variations to each view. In [40], S. Fujita et al. divided the high dimensional 4D light field into multiple 2D subspaces. Then, denoising was performed by cascading two or three CNNs applied to different subspaces.

In this work, leveraging the 3DFIS develop in our previous research [32], we further explore the adaptation of a convolutional neural network to multi-view denoising. We demonstrate that with the help of discriminative learning, denoising performance using CNN can be elevated to a higher level.

## 3. The Proposed Denoising Network

In this section, we present the proposed multi-view denoising network (MVCNN) that features multi-view input and output. The network architecture is modified from DnCNN [28], such that it can take a 3D matrix composed of multiple images as the input. In order to capture the inter-view image redundancy, we require the images in the input matrix to be well-aligned in the third dimension. We also adopt residual learning [41] and batch normalization [42], strategies that are popular in other computer vision tasks.

### 3.1. Network Architecture

Existing CNN models [27,28,29,30,31] are all designed exclusively for single image denoising. In the multi-view scenario, the most intuitive approach is to perform these single image algorithms on each of the views separately. This method, though simple and convenient, does not exploit the redundant image information that exists in multiple views capturing the same scene. Numerous studies [32,33,34,35,36,37,38,39,40,41,42,43,44] have demonstrated that inter-view redundant information is essential for recovering the original image details without creating undesirable over-smoothing artifacts that are common in single image denoising. Therefore, we believe that the denoising performance of CNN model can be further enhanced if inter-view information, in addition to intra-view information, is taken into consideration.

**Figure 1:**
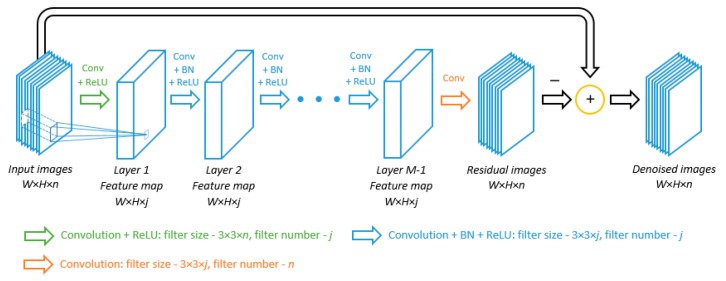
The architecture of the proposed network.

Distinct from the single image denoising network that takes single images or patches as the input, our proposed MVCNN accepts a 3D input matrix, which consists of multiple noisy images or patches. Figure 1 illustrates our proposed deep network, which is composed of *M* layers. Given *n* images of dimension *W* × *H* forming an input matrix of size *W* × *H* × *n*, the first layer consists of *j* convolution filters of size 3 × 3 × *n* and a rectified linear unit (ReLU) as the activation function that provides non-linearity. This result in an output of dimension *W* × *H* × *j*, which acts as the input of the next layer. For layer 2 to *M −* 1, there are three components including *j* convolution filters of size 3 × 3 × *j*, batch normalization and ReLU. The batch normalization is included to alleviate the effects of internal covariate shift [42] as well as to speed up the training process. The last layer contains *n* convolution filters of size 3 × 3 × *j* to generate an output that has the same dimension with the input of the network. In each layer, zero padding of length 1 is added before convolution so that the image dimension does not change as it passes through the network. Note that while the input images can be any size, the number of input images is fixed as *n*, since this determines the inner structure of the network, i.e., the filter size in the first layer and the number of filters in the last layer. If we have a different number of input images, we can either retrain the network or divide the input images into groups of *n* and integrate the denoised images of those groups.

Similar to previous CNN denoising models [28,29,30,31], we also adopt a residual learning [41] strategy by training a residual mapping that maps the noisy images to noise components. The clean images can then be obtained by subtracting the noise components from the noisy images. Previous research [28] has indicated that residual mapping is not only easier to be optimized but also helps batch normalization in reducing an internal covariate shift. Specifically, the loss function *l* on the network parameters Θ is defined as:

(1) $$l\left(\Theta \right)=\frac{1}{N_{s}}\sum_{i}d\left(R\left(Y_{i},\Theta \right),\;Y_{i}-X_{i}\right),$$

where *Y_i_* is the matrix of noisy image, *X_i_* is the matrix of ground truth image with *i* referring to the *i*th training sample, and *N_s_* is the number of training samples. In Equation (1), *R*(*Y_i_*, Θ) stands for the residual matrix that is mapped from *Y_i_* with network parameters Θ, and *d*(·,·) represents the distance between the estimated and ground truth residuals.

In implementation, we set the number of images in the input to be *n* = 9, which is common in multi-view imaging scenarios. In consideration of the tradeoff between complexity and performance, we set the number of layers *M* as 12. This number is sufficient to capture the inter-view dependencies for our multi-view denoising, while more layers will dramatically increase the computation burden. The number of feature maps *j* in each layer is dependent on the number of input images. More input images would not only require a larger number of feature maps, but also increase computational time. In our implementation, considering the tradeoff between performance and complexity, we empirically set *j* = 96. As for the distance metric *d*(·,·) in Equation (1), we will use the Euclidean distance.

In order to capture the pixel correlations across different views, during the training stage, we take a single image from the dataset and duplicate it multiple times to form the input 3D matrix. Additive white Gaussian noise is then added to the input matrix, and the corresponding output matrix is the noise matrix. In other words, all pixels in the input matrix can be considered well-aligned along the third dimension. More discussions of this kind of training input setting will be provided in Section 3.3, and detailed parameter settings of network training are described in Section 5.

### 3.2. Network Testing: Single Image vs. Multi-View

**Figure 2:**
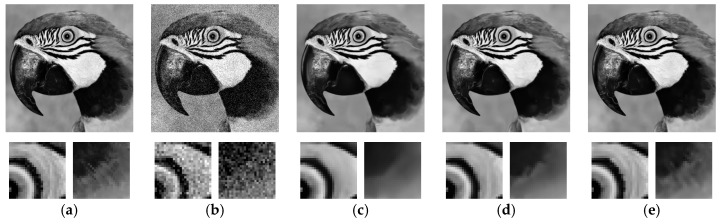
Comparison of single image convolutional neural network (CNN) and multi-view CNN models. (**a**) Ground truth; (**b**) Noisy image, PSNR = 20.15 dB; (**c**) DnCNN [28], PSNR = 29.44 dB; (**d**) Multi-view image denoising algorithm based on convolutional neural network (MVCNN) (3 views), PSNR = 31.91 dB; (**e**) MVCNN (9 views), PSNR = 34.68 dB.

In order to test the proposed network, we duplicate single images from the testing set multiple times such that they form 3D matrices with each pixel coordinate containing a vector of pixels having the same intensity value. Synthetic Gaussian noise is then added to the 3D matrices to form the noisy input. The trained network is then applied to the testing input matrices that are composed of well-aligned pixels. Since the network is trained in a way such that pixel correlations between well-aligned pixels are fully exploited, the output images from the network in these tests should be properly denoised without any artifacts. The denoising result for a different number of images (1, 3, and 9) involved in the input matrix with white Gaussian noise (*σ* = 25) added is shown in Figure 2. Two regions with fine textures are enlarged specifically for close inspection. As can be observed, with an increasing number of images in the input, the inter-view dependency can be better exploited, which leads to superior detail preservation than single image denoising. The peak signal-to-noise ratio (PSNR) comparison also indicates that the denoising performance improves with more views added to the input.

However, in multi-view denoising, the multiple views are not perfectly aligned due to different camera positions. Pixels from one view are moved by a certain distance, which is called disparity, in other views. The disparity is closely related to the depth of the surface point, in an inversely proportional manner. In order to align corresponding points from all views, we have previously introduced the 3D focus image stacks (3DFIS) [32] that generate several 3D image stacks consisting of translated multi-view images. Each stack corresponds to a specific disparity (or depth) value such that corresponding pixels with that disparity are located on the same coordinates. Therefore, regions with the correct disparity values appear to be well-aligned in the corresponding image stack and the proposed MVCNN can be properly employed. Details of the denoising algorithm that combines the network output with 3DFIS and the disparity map are elaborated in Section 4.

### 3.3. Signal Processing Interpretation of MVCNN

Referring to Figure 1, the MVCNN network produces residual images which resemble spatially uncorrelated Gaussian noise inherent in the input image. This seems to be quite different from popular CNN networks, where the output feature maps are used for detecting/classifying the content of the image. In this subsection, we will provide some signal processing interpretations of the function of the MVCNN.

#### 3.3.1. General Mechanism of the Denoising Network

**Figure 3:**
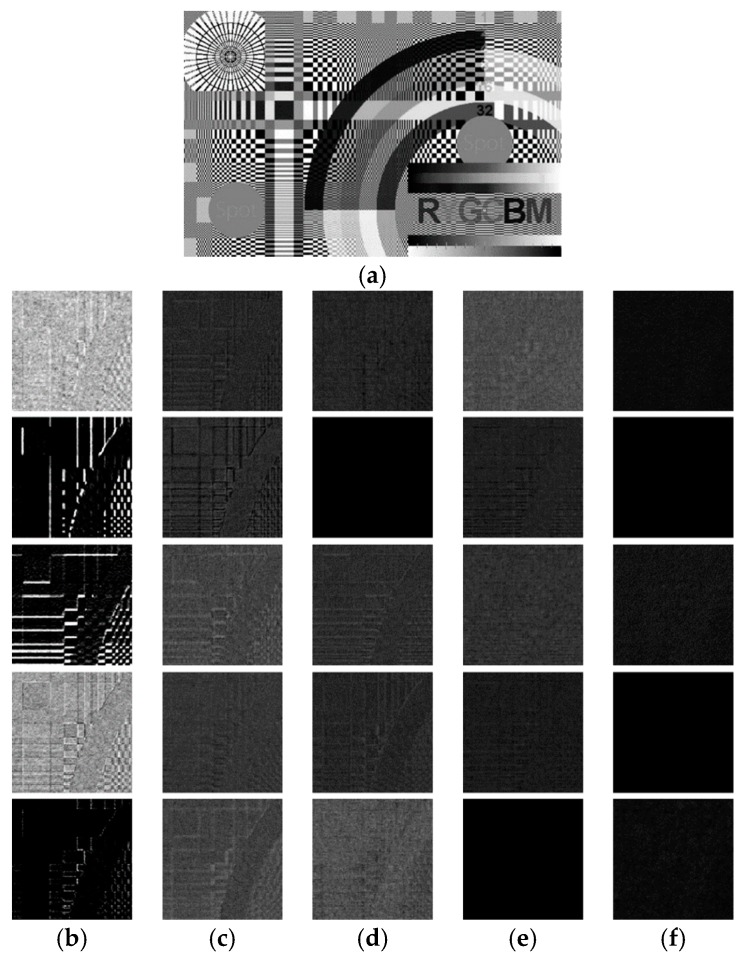
(**a**) Ground truth image; (**b**–**f**) Patches from first five feature maps from the output of Layer 1, 3, 5, 7, and 11 (*M* = 12).

To illustrate the mechanism behind the denoising network, we draw the feature maps generated in different layers as shown in Figure 3 using an image that consists of a wide range of frequency components. In the first layer, the convolution filters act as feature extraction operators to acquire numerous important features from the input images, such as edges, corners, and other textures, including noise. Then, in subsequent layers, the pixel correlations and dependencies in the images are exploited and removed from the feature maps. This results in a number of refined feature maps that are mostly comprised of noise components, which have no correlations among neighboring pixels. The final layer then reconstructs the residual images from the refined feature maps.

**Figure 4:**
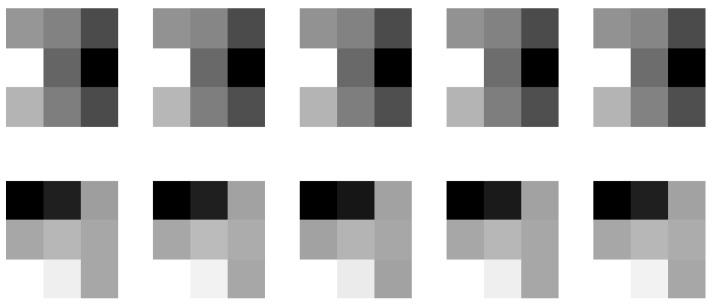
Parameters of the second (**top row**) and third (**bottom row**) convolution filters in the first layer (only the first 5 out of 9 matrices are displayed for each 3 × 3 × 9 filter, bright color indicates large values and dark color means small values).

To justify our conjecture, the output of the first layer is shown in Figure 3b. Due to the page limit, we only display the first five feature maps. It can be observed that some of the feature maps look like the result of edge detection, such as those in the second and third row. The filter parameters corresponding to these feature maps are shown in Figure 4. For simplicity, here we only show five out of the nine 3 × 3 matrices in each 3 × 3 × 9 filter. We observe that these matrices have a similar structure to common edge detectors, and they resemble each other within each 3 × 3 × 9 filter when the edge detection effect is obvious. Therefore, we believe that the convolution filters in the first layer act as feature extractors that extract various feature information from the input images, although some of the features may be too complex to be represented by human-designed detectors.

The subsequent layers, except the last one, are responsible for processing these features. In other image tasks such as classification, these low-level features are processed to form higher-level semantic representations so that the network is able to relate these representations to particular categories of objects. In our denoising task, on the other hand, the intra-view and inter-view correlations in these features are explored such that the information related to image structures is gradually suppressed, as shown in Figure 3c–f. Further analysis of these two types of correlation is discussed below.

#### 3.3.2. Intra-View Correlation

**Figure 5:**
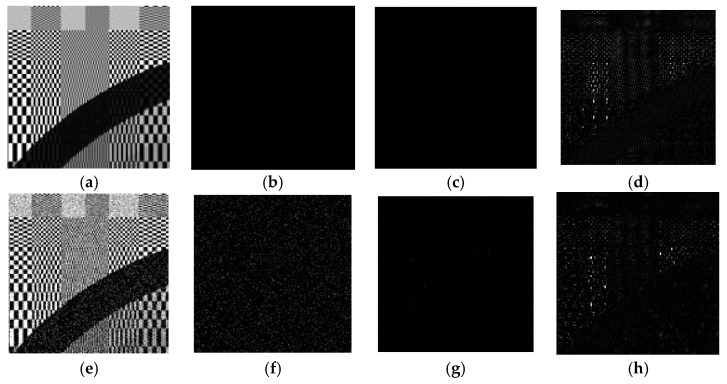
(**a**–**d**) Noise level = 0: input image, true residual image, estimated residual image, and estimated residual image with intensity rescaled to [0,15]; (**e**–**h**) Noise level = 25 and all input images have the same noise pattern: input image, true residual image, estimated residual image, and estimated residual image with intensity rescaled to [0,25].

During the processing of image features, pixel correlation within the image, which is also known as intra-view correlation, plays an important role in assisting the network to identify the image structures from the noise components. This correlation is also the foundation of most existing denoising algorithms. To further justify this claim, clean images without noise are sent into the network for testing. Theoretically, if the input images contain no noise, the estimated residual image should be all zeros. However, in reality, image components with very high frequency could demonstrate a bit of noise-like behavior due to the lack of sufficient pixel correlations within the neighborhood. This would lead to estimation errors in high-frequency regions. An example is shown in Figure 5a–d with regions of particularly high frequency being zoomed for inspection. Though hardly perceived by human eyes, estimation errors can be still observed in these high-frequency regions after rescaling the intensity.

#### 3.3.3. Inter-View Correlation

Apart from the intra-view correlations, the inter-view correlations are also essential to our multi-view denoising network. Since we use the same image and duplicate it multiple times to form a 3D input matrix during the training stage, so pixels at the same coordinates in different views are aligned in the input matrix. Therefore, there exists a strong correlation among pixels over the third dimension in the input matrix (before noise is added), and this correlation is called inter-view correlation. The network is trained to identify such correlation, in addition to intra-view correlation, so that image structure-related information can be further distinguished from noise information. This can be justified by a simple counterexample. If the additive noise has the same pattern in each image, then noise in each pixel is also correlated across different views, which will make the network misidentify the noise as image structures and hence incorrectly estimate the residual images. Figure 5e–h shows such an example by adding the same noise pattern to each of the input images. As a result, the estimated residual image shows significant bias from the true one, as if there is no noise.

### 3.4. Relationship with Previous Works

The proposed MVCNN shares a similar linear topology with some of the previously proposed deep networks. This linear topology, though simple, is very effective in performing various computer vision tasks, including denoising [27,28,29], super-resolution [45,46,47,48], image recognition [49,50], etc. Since the output of the network has the same dimension as the input, the pooling layer is typically not needed, and zero padding is often required in image denoising tasks. In comparison with DnCNN, which is the current state-of-the-art Gaussian denoiser for single images, our proposed network has a few similarities and some noticeable differences, detailed below.

**Figure 6:**
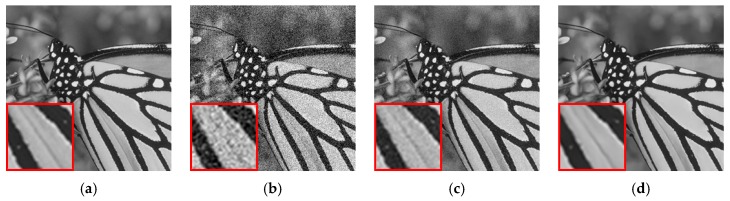
Denoising image (9 views) using different number of feature maps: (**a**) Ground truth; (**b**) Noisy image, 20.25 dB; (**c**) 64 feature maps, 29.71 dB; (**d**) 96 feature maps, 36.05 dB.

- The original DnCNN only takes one single image, which is a 2D matrix, as input for grayscale image denoising, while the input of MVCNN usually has a 3D input and output. This changes the filter size in the first convolution layer and the number of filters in the last layer.
- As the input matrix has more dimension, the number of layers and the number of feature maps also need to be adjusted accordingly. In specifics, the number of feature maps in each layer needs to be increased in order to capture sufficient inter-view correlations and achieve a satisfactory denoising performance. Figure 6 illustrates a denoising example using different numbers of feature maps. Meanwhile, as the number of feature map and number of views increment, the training time also rapidly increases. In order to keep a balance between denoising performance and computational complexity, we choose to slightly decrease the number of layers without sacrificing the performance.
- Simply passing the 3DFIS into the network produces a number of denoised image stacks, which are not the desired final denoised images. Further processing needs to be carried out to integrate these denoised image stacks into denoised images, with careful handling of occlusion. Therefore, a novel image fusion procedure and occlusion handling technique are proposed.

## 4. Multi-View Denoising Algorithm

In this section, we describe, in more detail, the procedures of our multi-view denoising algorithm using the proposed MVCNN model. In general, we assume the multi-view images are acquired from a planar camera array in which the cameras are separated with equal distances. Each camera corresponds to a coordinate (*s*, *t*) ∈ Z^2^ on the camera array, and without loss of generality, we assume the center view (0,0) is the target view we want to denoise. Each noisy image *I_s_*_,*t*_ can be represented as:

(2) $$I_{s,t}\left(x,y\right)=I_{s,t}^{\prime }\left(x,y\right)+n_{s,t}\left(x,y\right),$$

where *x* and *y* are pixel coordinates in each image, *I’_s_*_,*t*_ is the ground truth clean image and *n_s_*_,*t*_ is the i.i.d. zero-mean Gaussian noise with variance *σ*^2^. The objective is to estimate the clean target image from the multiple noisy images.

**Figure 7:**
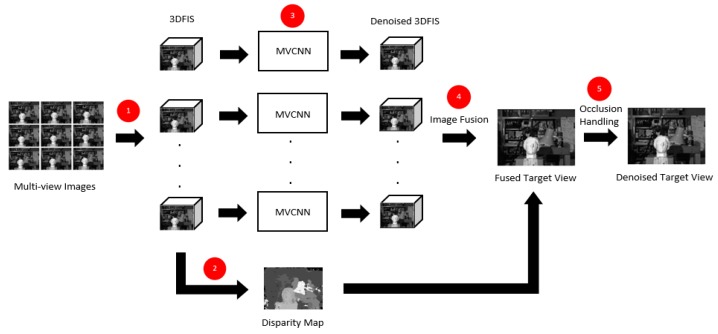
Processing pipeline of the proposed denoising algorithm: (**1**) Construct the 3D focus image stacks (3DFIS); (**2**) Estimate the disparity map; (**3**) Process the 3DFIS using the MVCNN model; (**4**) Image fusion from the denoised 3DFIS and disparity maps; (**5**) Occlusion detection and handling.

The general procedure of the proposed multi-view denoising algorithm is summarized as follows. First, the multi-view images are transformed into a number of 3D focus image stacks with respect to different disparity values, and the disparity map for the target view is estimated. Next, each of the image stacks is processed by the MVCNN model to remove the noise. Finally, the final denoised image is estimated by extracting and fusing corresponding in-focus regions from each of the denoised image stacks using disparity values. The processing pipeline of the algorithm is summarized in Figure 7, and the details of the algorithm are discussed in the following subsections.

### 4.1. 3D Focus Image Stacks and Disparity Estimation

In our previous work [32], we have introduced the notion of 3D focus image stacks (3DFIS) and utilized 3DFIS as an efficient way of searching for similar patches. The proposed denoising algorithm also takes advantage of the merits of 3DFIS for the purpose of aligning corresponding pixels. Instead of searching for similar patches as we did in the previous work, we directly use the 3DFIS for denoising purpose. Assume that all the images have been rectified so that their epipolar lines are parallel to horizontal lines. This turns the complex homography between different views into pure translation, which significantly simplifies the problem.

For a number of candidate disparity values *d* = 1, …, *d_max_*, we create a series of image stacks *F^d^* by translating the views and stacking them into 3D matrices as:

(3) $$F^{d}\left(x,y,k\right)=I_{s,t}\left(x-s\cdot d,y-t\cdot d\right),\;d=1,\;\ldots ,\;d_{max}$$

where *k* is an integer that has a unique mapping to each of the camera coordinates (*s*,*t*). From the perspective of stereo vision, for a pixel (*x*,*y*) in the target image, its corresponding image coordinate in other views will be of a distance away from (*x*,*y*) coordinates. Such distance is proportional to the disparity *d* of pixel (*x*,*y*). For example, corresponding points of pixel (*x*,*y*) in adjacent views are *d* pixels away from (*x*,*y*) in either horizontal or vertical directions, depending on the relative location of the view with respect to the target view. However, if a view is not adjacent to the target view, but is separated by a number of other views (which can be found using the camera coordinates (*s*,*t*)), then the corresponding points of (*x*,*y*) will be *s*·*d* and *t*·*d* away instead.

Therefore, if a pixel (*x*,*y*) in the target view has a true disparity *d*, its corresponding points in other views, which have distances of *s*·*d* and *t*·*d* in the *x* and *y* directions, will be shifted to the same position in the image stack *F^d^*. In other words, all pixels with disparity value *d* will be well-aligned (which is called in-focus) in *F^d^*. Since we have trained the MVCNN such that the network is able to denoise pixels that are well-aligned. Applying MVCNN model to each *F^d^* will remove the noise in regions that have true disparity *d*. By going through all the 3DFIS *F^d^* (*d* = 1, …, *d_max_*) using the denoising network, we obtain a series of image stacks with different in-focus regions being denoised, which will be elaborated in the next subsection.

The disparity map, which is the key to fuse the denoised 3DFIS into the final denoised image, can be estimated from the 3DFIS using photo-consistency between different views. Previously, we have proposed a robust disparity map estimation algorithm [32] that achieves a satisfactory error rate under noise interference. In this work, we use the same algorithm with a few modifications to obtain the disparity map. In specific, after obtaining the cost function *C*(*x*,*y*,*d*) for each pixel (*x*,*y*) and for each candidate disparity *d*, we further append a smoothness term *S*, which computes the sum of absolute differences of disparity values of adjacent pixels. The goal is to minimize the following objective function

(4) $$E=\sum_{\left(x,\;y\right)}C+\lambda \sum_{\left(x,y\right)}\sum_{\left(i,j\right)\in N_{4}\left(x,y\right)}S\left(i,j\right),$$

where *C* is the cost function we defined previously in [32] as

(5) $$C\left(x,y,d\right)=\frac{1}{n_{p}K}\sum_{k=1}^{K}\sum_{\left(i,j\right)\in W\left(x,y\right)}\left|F^{d}\left(i,j,k\right)-I_{0,0}\left(i,j\right)\right|,$$

*S* serves as the smoothness term which is defined as

(6) $$S\left(i,j\right){|}_{\left(i,j\right)\in N_{4}\left(x,y\right)}=\left|d\left(i,j\right)-d\left(x,y\right)\right|,$$

and *λ* is a weighting coefficient that balances the cost function and smoothness term. In Equation (5), *W*(*x*,*y*) is the patch centered at (*x*,*y*), *n_p_* is the number of pixels in each patch, and *K* is the number of views. In Equation (6), *N*_4_(*x*,*y*) is the four-neighborhood of pixel (*x*,*y*), and *d*(*x*,*y*) is the estimated disparity value for (*x*,*y*). The final disparity map can be estimated by optimizing Equation (4) using graph cut [51].

Note that what we have described so far in this subsection is the only part that has been introduced in our previous work [32]. The rest of the paper proposes a novel denoising method using the 3DFIS, disparity map and the trained MVCNN model.

### 4.2. Multi-View Denoising Using MVCNN

The denoising process involves processing 3DFIS *F^d^* by feeding *F^d^* into the proposed MVCNN model for each disparity *d* = 1, …, *d_max_*. According to our analysis in Section 3, the MVCNN model will generate a number of 3D matrices *R^d^* consisting of pure noise corresponding to each *F^d^*. Then the matrix of clean images ${\hat{F}}^{d}$ can be acquired by subtracting the noise matrix *R^d^* from the input image stack *F^d^*, i.e., ${\hat{F}}^{d}=F^{d}-R^{d}$. If all the images in *F^d^* are well-aligned, then ${\hat{F}}^{d}$ contains the images that have been denoised.

**Figure 8:**
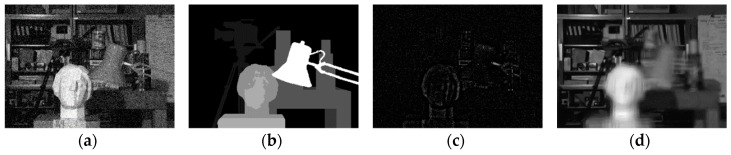
Denoising 3DFIS *F*^5^ using MVCNN. The background has the correct disparity *d* = 5. (**a**) Target noisy image; (**b**) true disparity map; (**c**) estimated noise components; (**d**) denoised target image in *F*^5^.

However, in multi-view scenarios, due to parallax, only parts of the input images that are in-focus are actually well-aligned in corresponding *F^d^*, so that the MVCNN model is only able to correctly estimate the noise in these regions. For out-of-focus regions, the noise estimation may not be accurate due to the violation of the alignment rule of MVCNN. Figure 8 shows an example of denoising one of the 3DFIS *F^d^* (*d* = 5) using MVCNN. The background of the scene has the true disparity 5 and thus will be well-aligned in the input matrix *F*^5^. As can be seen from Figure 8d, the network successfully removes the noise in those background regions but fails to correctly estimate the noise in other out-of-focus regions, leaving undesirable blurring artifacts. This issue can be overcome by selecting the appropriate in-focus regions from each 3DFIS ${\hat{F}}^{d}$ using the disparity map in a plane sweeping [38] manner, as discussed in the next paragraph.

After initial denoising using MVCNN, we obtain several denoised 3DFIS ${\hat{F}}^{d}$ (*d* = 1, …, *d_max_*) with in-focus regions recovered from noise corruption. The next step is to extract these in-focus regions and fuse them into the denoised image. Intuitively, for each pixel (*x*,*y*), its disparity value *d*(*x*,*y*) can be found in the disparity map, and we can get its denoised pixel value from the image stack ${\hat{F}}^{d}\left(x,y\right)$. The denoised image can be acquired by performing this operation on every pixel. However, such a pixel-wise processing of in-focus regions tends to cause seams at disparity discontinuities, especially when the disparity value is not accurate. Although we have substantially improved the disparity accuracy in noisy conditions [32], estimation error in specific regions like flat areas and object boundaries are still inevitable. In response, we decide to adopt a patch-wise selection and aggregation strategy. For each pixel, we extract the patch centered at it from the denoised 3DFIS ${\hat{F}}^{d}$ and assign it to the corresponding position in the denoised image. Each pixel in the denoised image will then be covered by multiple patches, and we can take a weighted average of these patches to get the final pixel value. The weight depends on the difference between each patch *P* and the reference patch *P_ref_* as $w=e^{-{\left(P\;-\;P_{ref}\right)}^{2}}$. The weighted averaging scheme helps mitigate the impact of inaccuracy of disparity estimation.

Theoretically, all the views in the camera array can be denoised, as our MVCNN model generates the denoised image stack that consists of multiple shifted views. In this paper, without loss of generality, we will be focusing on denoising the target view for simplicity. The overview of the entire denoising algorithm is listed in Algorithm 1.

**Algorithm 1** Multi-view Image Denoising**Input**: Multi-view images *I_s_*_,*t*_, maximum candidate disparity value *d_max_*, pre-trained MVCNN, target image number *k*.**Output**: Denoised target image *I_est_*.**Initialize**: Denoised target image *I_est_* = zeros(size(*I_s_*_,*t*_)), weight matrix *W* = zeros(size(*I_s_*_,*t*_)).1: **for**
*d* = 1:*d_max_*2:  Construct 3D focus image stacks *F^d^* using Equation (3);3:  Obtain denoised image stacks ${\hat{F}}^{d}$ by applying MVCNN to *F^d^*;4: **end**5: Estimate the disparity map for the target image using Equations (4)-(6);6: **for** each pixel (*x*, *y*)7:  Find its disparity *d*(*x*, *y*);8:  Obtain a patch *P* centered at (*x*, *y*) in the kth image of image stack ${\hat{F}}^{d}\left(x,y\right)$, and compute  its weight w.r.t. the reference patch *P_ref_* as $w=e^{-{\left(P\;-\;P_{ref}\right)}^{2}}$;9:  Update *I_est_* = *I_est_* + *w*·*P*;10:  Update *W* = *W* + *w*;11: **end**12: Compute the denoised target image *I_est_* = *I_est_/W*;13: Detect and handle occlusion using Algorithm 2.

### 4.3. Occlusion Detection and Handling

Due to occlusion, the denoised image acquired from the above procedures still has blurring artifacts near object boundaries where disparity discontinuity occurs. This is caused by the inconsistent image contents in such regions as the surface points in the scene are only visible to part of the views. In these regions, it is not possible to find a 3DFIS in which the pixels are well-aligned, and thus the MVCNN tends to produce a blurry effect that is similar to the averaging different values.

**Figure 9:**
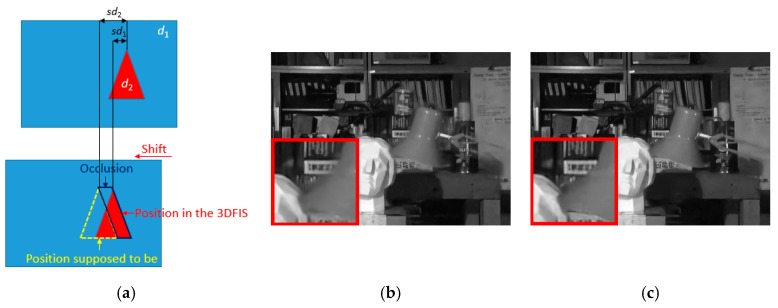
(**a**) Illustration of occlusion detection; (**b**) denoised image before occlusion handling; (**c**) denoised image after occlusion handling.

To handle the occlusion problem, we introduce a novel yet simple approach that estimates the occlusion regions using disparity values. Figure 9a illustrates the theory behind the detection algorithm. Suppose an image contains the background (blue) with disparity *d*_1_ and foreground object (red) with disparity *d*_2_, where *d*_2_ > *d*_1_. When we construct the 3DFIS, all pixels in the image are shifted by the same amount, e.g., by *s*·*d*_1_ (or *t*·*d*_1_ if shifting in the vertical direction), such that the background can be well-aligned in the corresponding image stack *F^d^*^2^. However, the foreground object should actually be shifted by *s*·*d*_2_ if we want to align them. Consequently, the difference, as indicated by the dark blue region in Figure 9a, is the occluded region that will appear as blurring artifacts after preliminary denoising. The occlusion amount can be computed as *s*·(*d*_2_ – *d*_1_) (or *t*·(*d*_2_ – *d*_1_) for vertical translations).

**Figure 10:**
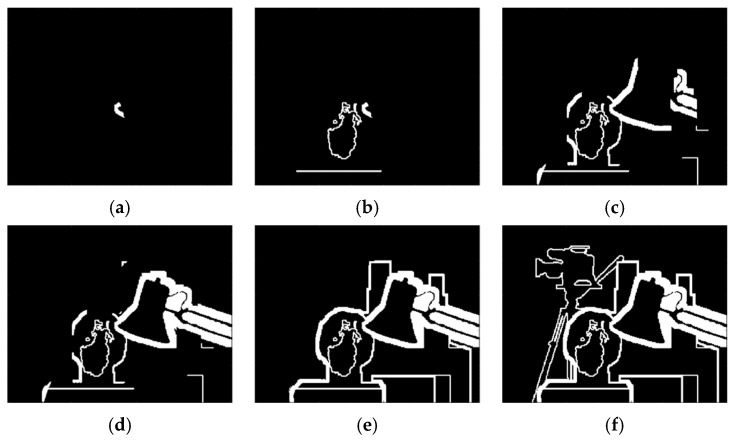
Occlusion detection incremental results: (**a**) iteration 4; (**b**) iteration 5; (**c**) iteration 7; (**d**) iteration 9; (**e**) iteration 10; (**f**) iteration 11.

In Algorithm 2, we propose an occlusion detection algorithm using this occlusion amount. Starting from the second closest objects with *d_curr_* = *d_max_* − 1 where *d_curr_* is the current disparity value, the region of these objects is selected, and a dilation operation is performed on regions of previous disparities with *d_prev_* > *d_curr_*. The kernel size of dilation is defined as twice the occlusion amount since we want to dilate symmetrically on both directions for each occluded pixel. Since we assume the target view is located on camera coordinate (0, 0), the kernel size of dilation *SE* can be simply defined as

(7) $$SE=\left(d_{prev}\;-\;d_{curr}\right)\cdot \left(s_{2}\;-\;s_{1}\right)+1,$$

where *s*_1_ and *s*_2_ are horizontal coordinates of the leftmost and rightmost cameras in the multi-view camera array. The vertical coordinates *t*_1_ and *t*_2_ of the top and bottom cameras can also be used and should lead to the same result for square camera arrays. In other words, the bigger the difference, the more dilation it requires as more areas will be occluded. In the case of non-square camera arrays, the larger one of *s*_2_ – *s*_1_ and *t*_2_ – *t*_1_ will be used in Equation (7). Next, we perform an AND operation of the current object regions and the dilated regions of previous disparities to get the occluded regions of the current objects. This procedure continues until we reach the minimum disparity *d* = 1. Figure 10 shows the incremental occluded regions estimated using Algorithm 2 for a sample disparity map. We can see that this algorithm efficiently captures the location and coverage of each occlusion. For pixels in the occluded regions, we simply denoise them using single image denoising methods, such as DnCNN. The effects of occlusion handling on removing the blurring artifacts are shown in Figure 9b,c.

In implementation, we empirically found that the MVCNN model can actually handle small amount of misalignment (e.g., 1–2 pixels) and still produce excellent denoising result that is better than its single image counterpart. Therefore, we further apply morphological transformations using erosion and dilation to eliminate small occlusions. Given the occlusion map we get from Algorithm 2, an image erosion, followed by an image dilation with the same kernel size, is performed in sequence.

Meanwhile, when the images are seriously corrupted by noise, the disparity estimation can be much less accurate, resulting in an overwhelming number of false positives in occlusion detection. Moreover, single image denoising, including state-of-the-art methods like DnCNN, tends to create significantly blurry artifacts at high noise levels. These two factors combine to make the occlusion handling unreliable when the noise is high. In such cases, we determine to refine the occlusion map using edge detection. When the noise level *σ* ≥ 30, the edge map of the image was estimated using the Canny edge detector, such that only significantly noticeable edges are detected. Both the occlusion map and the edge map are dilated to increase their compatibility and robustness. Finally, we perform an AND operation on the edge map and occlusion map to eliminate the false positives. This process helps suppress blurry artifacts by strictly limiting the regions of single image denoising replacement only to those with a large number of misalignments.

**Algorithm 2** Occlusion Detection**Input**: Disparity map *D*.**Output**: Occlusion map *O*.**Initialize**: *O* = zeros(size(*D*)).1: **for**
*d_cur_* = (*d_max_* – 1):−1:12:  *A_cur_* = {(*x*, *y*): *D*(*x*, *y*) = *d_cur_*};3:  **for**
*d_prev_* > *d_cur_*4:     *SE* = (*d_prev_* – *d_cur_*)(*s*_2_ – *s*_1_) + 1;5:     *A_prev_* = {(*x*, *y*): *D*(*x*, *y*) = *d_prev_*};6:     *A_dilate_* = imdilate(*A_prev_*, *SE*);7:     *O* = (*A_cur_* & *A_dilate_*) | *O*;8:  **end**9: **end**

## 5. Experimental Results

### 5.1. Parameter Settings for Network Training

For the purpose of training the network, we used a dataset consisting of 68 natural images from Berkeley segmentation dataset [52]. Since our input contains 9 images, it is equivalent to 612 images for training in single image denoising. Adding more images does not empirically improve the denoising performance significantly, but tremendously increases the training time and computer memories. Patches of size 40 × 40 are extracted from each image with a stride of 10 pixels. Each patch is then duplicated 9 times and stacked into a 3D matrix with AWGN of noise level *σ* = 15, 25, 35, 50 being added. Each image in the dataset has a dimension of 481 × 321, thus creating a total of 1536 × 612 patches for network training.

The noisy 3D matrices, as well as their ground truth, are fed into the network to learn the weights of convolution layers. The loss function *l* defined in Equation (1) is optimized using the Adaptive Moment Estimation (Adam) algorithm [53]. The mini-batch size is 128, and we train the MVCNN model for 50 epochs. The learning rate decreases from 10^−3^ to 10^−4^ as the training errors drop along the training process. The MVCNN model is trained in Matlab R2018a environment with MatConvNet package [54] on a PC with Intel^®^ CoreTM i7-6700K CPU 4GHz and Nvidia GeForce^®^ GTX 980 Ti GPU. The whole training process takes around 6–7 h for grayscale images, and 12–14 h for color images on GPU.

**Figure 11:**
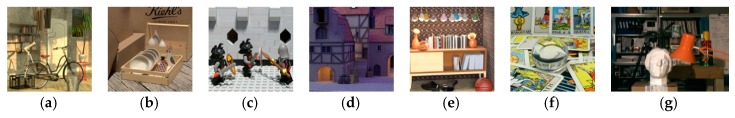
Testing image datasets: (**a**) Bicycle; (**b**) Dishes; (**c**) Knights; (**d**) Medieval; (**e**) Sideboard; (**f**) Tarot; (**g**) Tsukuba.

The datasets that we use to evaluate the denoising algorithm consist of seven multi-view image sets from different online datasets, as shown in Figure 11. The “Tsukuba” dataset is from the Middlebury multi-view stereo dataset [55]. The “Knights” and “Tarot” datasets are from the Stanford light field archive [56]. The “Bicycle”, “Dishes”, “Medieval”, and “Sideboard” datasets are from the 4D light field benchmark [57]. For all image datasets, we take a subset of nine images (3 × 3) for our experiment, and all the images except “Tsukuba” are resized to 256 × 256 for the purpose of simplicity and efficiency.

### 5.2. Blind Denoising

In most image denoising literature, including our proposed MVCNN model, it is assumed that the noise level is already known so that the algorithm can be applied using a specific noise variance *σ*^2^. This requires that the noise level should be pre-estimated if images of unknown noise are given, which makes the denoising performance affected by the accuracy of noise estimation. In the case of Gaussian noise of unknown variance, instead of estimating the noise level, we train the network using images with a wide range of noise levels. Specifically, different levels of noise (e.g., *σ* ∈ [0,55]) are added to different layers of the input 3D matrix, with σ remains the same within each layer. The CNN model trained in this way is capable of handling images with various noise levels. With this blind denoising scheme, we no longer need to train several networks with respect to different noise levels. As long as the noise level of test images is within the range of [0,55], the proposed denoising model can still estimate the clean image without knowing the noise variance. We refer to this blind denoising model as MVCNN-B.

### 5.3. Color Image Denoising

The size of input color images is set to *W* × *H* × 3, where 3 denotes the RGB channels. The network described in Section 3 is modified such that the input of the network has a dimension of *W* × *H* × 3*n*, where n is the number of views. Specifically, the convolution filters in the first layers now have the dimension of 3 × 3 × 3*n*, and the number of filters in the last layer is 3*n*, so that the output has the same dimension of input. The training parameters remain the same as grayscale image denoising. Likewise, the 3DFIS also has shifted images of all RGB channels, which makes each stack three times thicker. All other procedures are the same as grayscale image denoising. We refer to the color image denoising model as MVCNN-C.

### 5.4. Evaluation of Denoising Performance

We compare our proposed MVCNN and MVCNN-B methods with existing state-of-the-art denoising algorithms, including both single image and multi-view denoising. In comparison with single image denoising, we experimented on BM3D [6], WNNM [8], and DnCNN [28]. The first two are representative methods that explore the non-local self-similarity image prior, while the last one is one of the more popular algorithms in discriminative learning. For a multi-view denoising comparison, we employed three algorithms that demonstrate decent denoising performance, including Miyata’s fast denoising algorithm [37], VBM4D [58] and our previous work [32] (Zhou et al.). VBM4D is an extension of BM3D that handles volumetric data using 3D or 4D input images or videos. When applied to our multi-view scenario, the multiple views can be stacked into a 3D matrix and fed into the algorithm. Our previous work has successfully denoised image by exploring non-local self-similarity, both within the target view and across other views, and exhibited comparable or even better performance than VBM4D.

For color image denoising, since some of the denoising algorithms do not support color images, we compare the proposed MVCNN-C method with CBM3D [59], CDnCNN [28], and CVBM3D [59] algorithms. CBM3D and CDnCNN are just color versions of the BM3D and DnCNN methods. CVBM3D is an RGB video denoising algorithm that can also be applied to multi-view images by treating the images as a sequence of frames.

Table 1 shows the PSNR values of different methods on various datasets for grayscale image denoising. As can be observed, the three single image denoising methods have relatively similar denoising performance, with WNNM and DnCNN outperforming BM3D by a little margin. On the other hand, benefiting from inter-view image redundancies, multi-view denoising algorithms exhibit considerably enhanced performance for most of the datasets. Our previous work has been consistently outperforming single image denoising by around 1–2 dB across all noise levels. The VBM4D method also shows excellent denoising performance when the disparity values between different views are small but falls behind if adjacent views have a large disparity, such as the “Tsukuba” dataset. The method of Miyata et al. exhibits satisfactory denoising performance under low-level noise, but the quality of the denoised image quickly deteriorates as the noise level increases due to its oversimplified nature. Nevertheless, the proposed MVCNN and MVCNN-B excel these competing multi-view denoising algorithms by a margin of around 1–2 dB, especially when the noise level is high. The fixed noise model MVCNN slightly outperforms the blind model MVCNN-B, which is expected since the fixed noise model is able to explore the noise characteristics when all training samples have the corresponding noise level.

**Figure 12:**
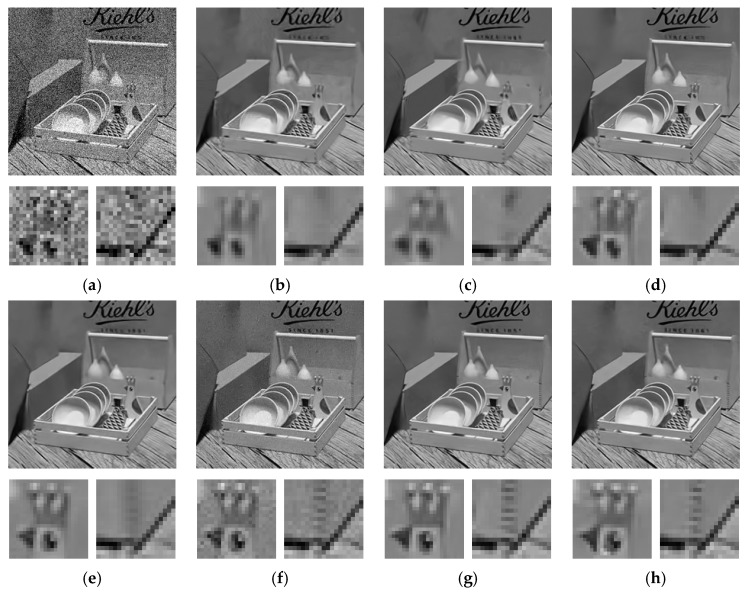
Denoising performance of different methods on “Dishes” dataset with noise level *σ* = 25. (**a**) Noisy image; (**b**) BM3D [6]; (**c**) WNNM [8]; (**d**) DnCNN [28]; (**e**) VBM4D [58]; (**f**) Zhou et al. [32]; (**g**) MVCNN; (**h**) MVCNN-B.

**Figure 13:**
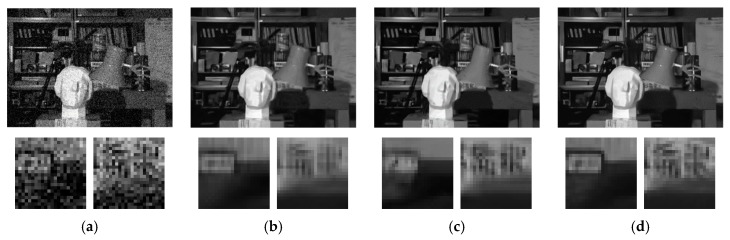

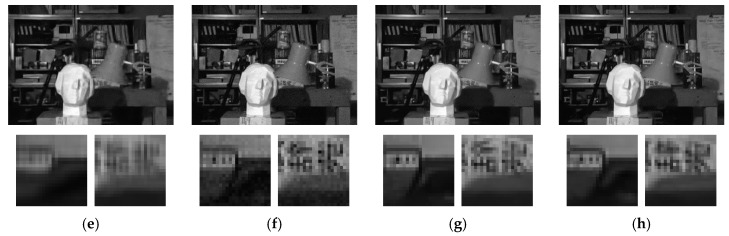
Denoising performance of different methods on “Tsukuba” dataset with noise level *σ* = 25. (**a**) Noisy image; (**b**) BM3D [6]; (**c**) WNNM [8]; (**d**) DnCNN [28]; (**e**) VBM4D [58]; (**f**) Zhou et al. [32]; (**g**) MVCNN; (**h**) MVCNN-B.

The visual results of different methods are illustrated in Figure 12 and Figure 13. Two regions are zoomed in so that the comparison of details can be closely observed. From the visual comparison, we can see that single image denoising algorithms, including BM3D, WNNM, and DnCNN, tend to over-smooth find details such as edges and textures. VBM4D exhibits severe ghost artifacts if the disparity is large between different views as shown in Figure 13e. Our previous work is able to preserve those details, but at the cost of keeping some of the noise in the estimated image. This results from the principle of the algorithm that is heavily dependent on the number of views, and the issue can be mitigated by including more views into the denoising. In comparison, the proposed MVCNN and MVCNN-B demonstrate significantly more consistent and reliable denoising performance with preservation of fine details. The fixed noise model and blind model do not have an observable difference in terms of visual appearance.

Table 2 shows the color image denoising performance of different methods when the noise level is 25. Similar to grayscale image denoising, our proposed network significantly outperforms the two comparing single image denoising algorithms (CBM3D, CDnCNN), and exhibits a competitive performance with CVBM3D on most datasets with an average PSNR lead of 0.41 dB. Note that although CVBM3D obtains slightly better PSNR on some of the datasets, it suffers from the same problem of large disparities (e.g., the “Tsukuba” dataset) as its grayscale version, VBM4D, while MVCNN-C demonstrates a more consistent denoising performance so that it can be applied to more general situations.

**Figure 14:**
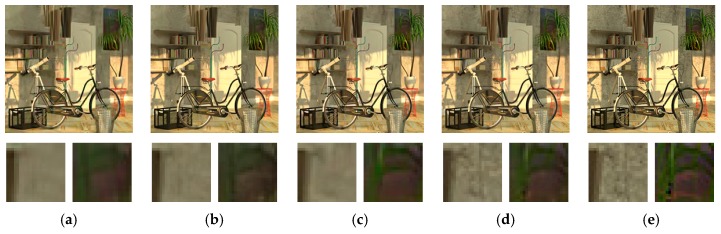
Color image denoising performance on “Bicycle” dataset with noise level *σ* = 25. (**a**) CBM3D [59]; (**b**) CDnCNN [28]; (**c**) CVBM3D [59]; (**d**) MVCNN-C; (**e**) Ground truth.

**Figure 15:**
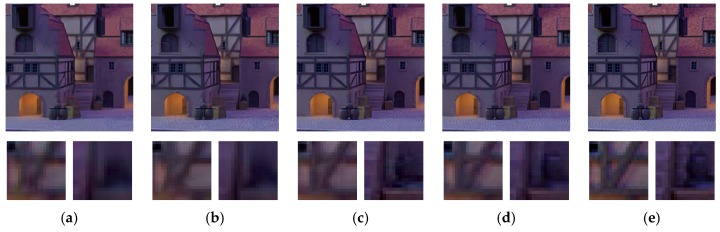
Color image denoising performance on “Medieval” dataset with noise level *σ* = 25. (**a**) CBM3D [59]; (**b**) CDnCNN [28]; (**c**) CVBM3D [59]; (**d**) MVCNN-C; (**e**) Ground truth.

Figure 14 and Figure 15 illustrate the visual quality of different methods on color images. As we can see, both CBM3D and CDnCNN tend to over-smooth the image structures, making them visually unrecognizable. In comparison, the multi-view methods, CVBM3D and MVCNN-C, present great detail preservation and superior denoising performance. In particular, when the ground truth image contains noise-like textures, such as the “Bicycle” dataset (Figure 14), our proposed MVCNN-C is still able to separate the noise from the texture without creating blurring artifacts, while the other three comparing methods failed to do so.

### 5.5. Run Time

Given multi-view images with a size of *W* × *H*, the complexity of the proposed denoising algorithm using trained MVCNN is O(*W*·*H*·*K*·*M*·*j*), where *K* is the total number of views, *M* is the number of layers in the network, and *j* is the number of feature maps in each layer. The occlusion detection and patch aggregation processes are negligible compared to the convolution computation. In comparison, our previous approach [32] is a patch-based denoising algorithm with the complexity of O(*W*·*H*·*K*·*n*·*r*^2^·*R*^2^) for the patch matching procedure and O(*W*·*H*·[(*r*^2^)^2^·*K*·*n* + (*K*·*n*)^3^]) for the SVD operation [60], where *n* is the number of similar patches, *r* and *R* are side lengths of patches and searching windows, respectively.

Table 3 lists the runtime of different algorithms on various datasets (grayscale) with noise level 25. The “Tsukuba” dataset contains images with a size of 288 × 384, while images in all other datasets are of size 256 × 256. BM3D and VBM4D are written in C/C++ and called in Matlab using the MEX functions. The other algorithms are written in Matlab. The memory transfer time between CPU and GPU is counted for DnCNN and our MVCNN algorithm.

From the table, we can observe that the DnCNN using GPU is the fastest method of all competitors, which is understandable due to the fast inference of the convolution neural network. Furthermore, the deep learning package MatConvNet [54] is a fully optimized library that is more efficient than our hand-written code. When just comparing with multi-view denoising algorithms, our proposed MVCNN is around ten times faster than VBM3D and takes much less time than our previous work. This is also within expectation, since the proposed method does not involve the time-consuming patch matching and SVD operations. Specifically, for MVCNN, most of the time is spent on the construction of the 3DFIS, as we have many more dimensions in multi-view denoising than single image denoising, including the number of views and disparity values. The actual denoising time of MVCNN on each image stack is comparable to that of DnCNN. Therefore, we believe that, like DnCNN, with the optimization of image stack constructions, our proposed MVCNN shows a more promising prospect in real life applications than other comparable approaches.

## 6. Conclusions

In this paper, we proposed a new CNN model, namely MVCNN, for multi-view image denoising. Unlike single image CNN models, the proposed network can take multiple images formed as a 3D matrix as the input and produce a denoised 3D matrix consisting of clean images. The 3D focus image stacks introduced in our previous work are generated from multiple views to form inputs to the MVCNN network, and disparity values are utilized to extract the corresponding denoised parts in each image stack. Extensive experiments that we have performed indicate that the proposed MVCNN model produces a state-of-the-art performance for image denoising. Meanwhile, compared to existing multi-view denoising algorithms, MVCNN also achieves faster computational speed thanks to the fast inference of convolutional neural network and GPU acceleration. In the future, we will be focusing on the denoising of real image noise, which is more complicated than AWGN.

## Figures and Tables

**Table 1:** Grayscale image denoising performance (PSNR—dB) comparison of different methods on various datasets with noise level 15, 25, 35, 50. (**bold**—best result, underline—second best result).

	BM3D [6]	WNNM [8]	DnCNN [28]	Miyata et al. [37]	VBM4D [58]	Zhou et al. [32]	MVCNN	MVCNN-B
*σ* = 15								
Bicycle	29.44	29.72	30.21	29.90	31.56	31.32	**32.10**	31.33
Dishes	31.49	32.34	32.56	31.03	33.65	33.54	**35.00**	34.78
Knights	30.97	31.80	31.91	30.73	33.51	33.00	**34.15**	33.72
Medieval	32.17	32.55	32.64	31.22	33.92	34.26	**35.92**	35.07
Sideboard	29.32	30.61	29.70	29.91	31.48	31.50	**32.70**	32.20
Tarot	28.20	28.71	29.25	27.96	30.27	**30.58**	30.32	29.96
Tsukuba	32.77	33.14	33.34	30.35	32.64	34.34	**35.48**	34.96
*σ* = 25								
Bicycle	26.72	26.92	27.51	26.60	28.93	28.60	**30.15**	29.32
Dishes	28.55	29.29	29.73	26.94	30.75	30.46	**32.89**	32.19
Knights	27.71	28.56	28.59	26.99	30.42	29.97	**32.02**	31.25
Medieval	30.19	30.58	30.44	27.20	31.73	31.37	**33.31**	32.70
Sideboard	26.31	27.43	26.60	26.47	28.62	28.77	**30.63**	29.83
Tarot	24.87	25.30	25.99	25.16	27.43	27.93	**28.15**	28.01
Tsukuba	29.65	30.28	30.09	26.65	29.66	30.89	**32.31**	32.04
*σ* = 35								
Bicycle	24.93	25.24	25.69	24.15	27.24	26.93	**28.68**	27.88
Dishes	26.36	27.35	27.47	24.31	28.66	28.32	**31.00**	30.28
Knights	25.51	26.53	26.30	24.42	28.23	27.75	**29.96**	29.35
Medieval	28.77	29.18	28.90	24.63	30.23	29.36	**31.16**	31.07
Sideboard	24.32	25.25	24.63	23.94	26.49	26.95	**28.95**	28.17
Tarot	22.74	23.33	23.81	23.00	25.36	26.03	**26.99**	26.60
Tsukuba	27.56	28.48	27.69	24.20	27.54	28.69	29.32	**29.51**
*σ* = 50								
Bicycle	22.86	23.44	23.84	21.34	25.39	24.96	**26.28**	26.26
Dishes	24.18	25.37	25.41	21.50	26.37	26.05	**27.90**	27.89
Knights	23.14	24.41	24.08	21.61	25.81	25.25	**26.92**	26.94
Medieval	26.57	27.68	26.43	21.79	28.31	27.30	28.93	**29.00**
Sideboard	22.09	23.12	22.70	20.94	24.03	24.89	26.03	**26.25**
Tarot	20.53	21.42	21.83	20.34	22.96	23.87	**25.03**	24.83
Tsukuba	25.07	26.66	25.01	21.62	25.14	25.95	**26.83**	26.69

**Table 2:** Color image denoising performance (PSNR—dB) comparison of different methods on various datasets with noise level 25. (**bold**—best result, underline—second best result).

	CBM3D [59]	CDnCNN [28]	CVBM3D [59]	MVCNN-C
Bicycle	28.26	29.23	30.45	**30.51**
Dishes	30.73	31.89	33.16	**33.74**
Knights	29.96	30.89	**32.74**	32.40
Medieval	31.01	31.45	**33.41**	33.26
Sideboard	27.69	28.63	29.73	**30.82**
Tarot	26.87	28.15	28.66	**28.95**
Tsukuba	31.13	31.64	31.40	**32.68**
Average	29.38	30.27	31.36	**31.77**

**Table 3:** Run time (seconds) comparison of different methods on various datasets (grayscale) with noise level 25.

	BM3D [6]	WNNM [8]	DnCNN [28]	Miyata et al. [37]	VBM4D [58]	Zhou et al. [32]	MVCNN
Bicycle	0.5	76.1	0.040	2.74	18.8	93.1	1.73
Dishes	0.6	76.3	0.044	2.70	18.6	99.6	1.82
Knights	0.5	75.5	0.045	2.68	17.5	94.4	2.08
Medieval	0.6	75.9	0.040	2.57	18.1	94.6	1.88
Sideboard	0.5	75.3	0.040	2.73	17.4	90.4	1.70
Tarot	0.4	78.5	0.048	3.48	17.6	94.1	1.73
Tsukuba	1.0	134.1	0.050	4.49	38.6	170.7	2.62
Average	0.58	84.53	0.044	3.06	20.94	105.27	1.94
